# Protection against Fas-induced fulminant hepatic failure in liver specific integrin linked kinase knockout mice

**DOI:** 10.1186/1476-5926-10-11

**Published:** 2011-11-21

**Authors:** Shashikiran Donthamsetty, Wendy M Mars, Anne Orr, Chuanyue Wu, George K Michalopoulos

**Affiliations:** 1 Department of Pathology, University of Pittsburgh School of Medicine, Pittsburgh, PA, USA

**Keywords:** Integrin linked kinase, Jo-2, Apoptosis, ECM signaling

## Abstract

**Background:**

Programmed cell death or apoptosis is an essential process for tissue homeostasis. Hepatocyte apoptosis is a common mechanism to many forms of liver disease. This study was undertaken to test the role of ILK in hepatocyte survival and response to injury using a Jo-2-induced apoptosis model.

**Methods:**

For survival experiments, ILK KO and WT mice received a single intraperitoneal injection of the agonistic anti-Fas monoclonal antibody Jo-2 at the lethal dose (0.4 μg/g body weight) or sublethal dose (0.16 μg/g body weight). For further mechanistic studies sublethal dose of Fas monoclonal antibody was chosen.

**Results:**

There was 100% mortality in the WT mice as compared to 50% in the KO mice. We also found that hepatocyte specific ILK KO mice (integrin linked kinase) died much later than WT mice after challenge with a lethal dose of Fas agonist Jo-2. At sublethal dose of Jo-2, there was 20% mortality in KO mice with minimal apoptosis whereas WT mice developed extensive apoptosis and liver injury leading to 70% mortality due to liver failure at 12 h. Proteins known to be associated with cell survival/death were differentially expressed in the 2 groups. In ILK KO mice there was downregulation of proapoptotic genes and upregulation of antiapoptotic genes.

**Conclusions:**

Mechanistic insights revealed that pro-survival pathways such as Akt, ERK1/2, and NFkB signaling were upregulated in the ILK KO mice. Inhibition of only NFkB and ERK1/2 signaling led to an increase in the susceptibility of ILK KO hepatocytes to Jo-2-induced apoptosis. These studies suggest that ILK elimination from hepatocytes protects against Jo-2 induced apoptosis by upregulating survival pathways. FAK decrease may also play a role in this process. The results presented show that the signaling effects of ILK related to these functions are mediated in part mediated through NFkB and ERK1/2 signaling.

## Background

Programmed cell death or apoptosis is an essential process for tissue homeostasis. Hepatocyte apoptosis is a common mechanism to many forms of liver disease. It has been recognized to contribute to the pathogenesis of alcoholic liver disease, nonalcoholic steatohepatitis, viral hepatitis, cholestatic liver disease, and ischemia/reperfusion injury [1-4]. Apoptosis can be triggered by Fas receptor mediated signaling as well as different stimuli that provoke cell stress. All these stimuli converge at the activation of caspase 3 that leads to internucleosomal DNA degradation, chromatin condensation, cell shrinkage, and formation of small apoptotic bodies that are phagocytosed by neighboring macrophages [4]. The liver is very sensitive to Fas-induced apoptosis. Administration anti-Fas agonistic antibody Jo-2 to mice leads to rapid death of the animals due to fulminant hepatitis, mimicking certain forms of acute liver failure (ALF) in humans [5]. Fas (CD95/APO-1), a 43-kDa cell surface glycoprotein, belongs to the tumor necrosis factor receptor superfamily, and mediates apoptosis upon binding with its cognate ligand, or artificially with specific agonistic antibodies.

Communication between cells and the extracellular matrix (ECM) is achieved through integrins and the associated integrin proximal adhesion molecules. Through multiple protein-protein interactions and signaling events, these molecules transmit signals from the ECM to the interior of the cell and regulate many fundamental cellular processes. Integrin-linked kinase (ILK) is a β1- and β3-integrin-interacting cell matrix adhesion protein that has been shown to be crucial for a number of cellular processes such as survival, differentiation, proliferation, migration, and angiogenesis [6-8]. Previous studies in our lab have shown that acute elimination of ILK by injection of adenovirus expressing Cre recombinase in the tail vein of ILKflox/flox mice led to massive hepatocyte apoptosis [9]. Genetic ablation of ILK also results in some degree of apoptosis [10] but also to an enhancement of hepatocyte proliferation, suggesting that ILK might be playing a role in hepatocyte survival. This study was undertaken to test the role of ILK in hepatocyte survival and response to injury using a Jo-2-induced apoptosis model. Here we report that genetic ablation of ILK from hepatocytes protects from Jo-2 induced apoptosis due to upregulation of survival signaling mainly ERK and NFκB signaling.

## Methods

### Generation of liver specific ILK/liver-/- mice

ILK floxed animals were generated as described previously [10] and donated by Drs. René St. Arnaud (Shriners Hospital and McGill University, Montréal) and Shoukat Deodhar (British Columbia Cancer Agency and Vancouver Hospital, Jack Bell Research Center, Vancouver), and mated with AFP-enhancer-albumin-promoter-Cre-recombinase-expressing mice which were kindly provided by Dr. Klaus Kaestner (University of Pennsylvania). The off-spring were genotyped as described previously [11] and the ILK-floxed/floxed Cre-positive mice were considered to be ILK-knockout (ILK KO), while their Cre-negative siblings were used as controls. All animals were housed in the animal facility of the University of Pittsburgh in accordance with the guidelines of the Institutional Animal Use and Care Committee of the University of Pittsburgh.

### Induction of apoptosis

For survival experiments, male 30 week-old ILK KO (n = 10) and control mice (n = 10) received a single intraperitoneal injection of the agonistic anti-Fas monoclonal antibody Jo-2 (BD Pharmingen, San Diego, CA) at the lethal dose (0.4 μg/g body weight) or sublethal dose (0.16 μg/g body weight) diluted in sterile saline. The mice were monitored for up to 24 hours, and the time of death was recorded. The Fas injury model was induced in controls and ILK KO mice with a single intraperitoneal injection of Jo-2 at the dose of 0.16 μg/g weight. At the indicated time points (up to 12 hours) after Jo-2 injection, mice were sacrificed. Livers were snap frozen in liquid nitrogen or formalin-fixed and paraffin embedded for histopathological studies. All procedures performed on these mice were approved under the IACUC protocol and conducted according to National Institute of Health guidelines.

### Isolation, culture and treatment of mouse hepatocytes

Hepatocytes were isolated from male ILK KO and control mice as described previously [10]. Cells were plated onto collagen-coated 6-well dishes (type I collagen, Collaborative Biomedical, Bedford, MA) 5 × 10^5 ^cells per well. Cultures were maintained in minimal essential medium supplemented with 10% fetal calf serum, nonessential amino acids, 2 mM glutamine, and antibiotics (all from Invitrogen). After 2-h incubation medium was removed, and cells were refed the same medium with 0.5% fetal calf serum and incubated overnight. Apoptosis was induced in cultured mouse hepatocytes by treatment with 0.5 μg/ml anti-Fas antibody and 0.05 μg/ml actinomycin D as described before [12]. The effect of ILK deletion on Fas-mediated apoptosis was also tested in the presence of the extracellular-regulated kinase 1/2 inhibitor U0126 (20 μM, Cell Signaling), the phosphatidylinositol 3-kinase (PI3K) inhibitor LY-294002 (50 μM, Cell signaling) and NFκB peptide (30 μM, Calbiochem). Doses of the inhibitors and peptides were selected based on previous studies with isolated hepatocytes [13].

### Measurement of apoptosis

Apoptotic nuclei were detected by terminal deoxynucleotidyl transferase-mediated deoxyuridine triphosphate nick-end labeling staining using the ApopTag Peroxidase kit (Millipore, Billerica, MA). Activation of caspase 3/7 in cell lysates was detected using a commercially available kit (Promega, Madison, WI).

### Western blot analysis

Liver Homogenates were prepared as described previously [10]. The following primary antibodies were used in this study: rabbit anti-cleaved caspase 3, Rabbit anti-BAD and phospho BAD, Rabbit anti-Bcl-2, Rabbit anti-Bcl-xl, Rabbit anti phospho Akt (serine 473), Rabbit anti phospho ERK (Thr202/Tyr204), Rabbit cleaved PARP, Rabbit p65 (Cell Signaling Technologies, Danvers, MA), Mouse anti Fas (Santa Cruz) and mouse anti-β-actin (Chemicon, Temecula, CA). Donkey anti-rabbit and anti-mouse secondary antibodies were purchased from Jackson ImmunoResearch Laboratories (West Grove, PA) and were used at 1:50,000 dilutions.

## Results

### Effect of genetic ablation of ILK from hepatocytes on Fas-induced animal death and fulminant hepatitis

**Figure 1:**
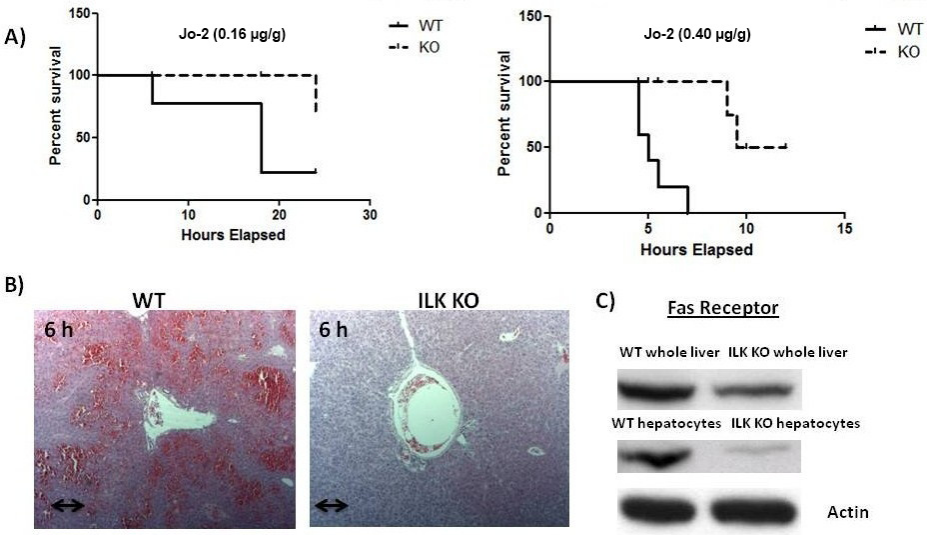
**Protection of ILK KO mice against Fas-induced liver injury and apoptosis**. **A) **Kaplan Meier survival curves after a sublethal (0.16 μg/g) (left graph) and a lethal dose (0.40 μg/g) (right graph) of Jo-2. **B) **Hematoxylin-eosin staining of liver sections at 6 h after a sublethal injection of Jo-2 shows reduced hemorrhage and apoptotic cell bodies in the ILK KO mice. Double arrow = 300 μm. **C) **Representative Western blots of basal levels of Fas receptor in whole livers and hepatocytes isolated from WT and ILK KO mice.

**Figure 2:**
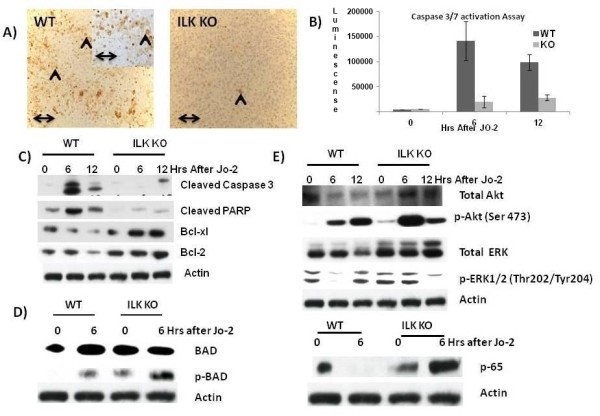
**Proteins associated with apoptosis and survival pathways are differentially expressed in the ILK KO mice**. **A) **Tunnel assay 6 h after a sublethal dose of Jo-2 showing increased number of apoptotic bodies in the WT mice as compared to the ILK KO mice. Double arrow = 300 μm. **B) **Caspase 3/7 activation after a sublethal dose of Jo-2. **C) **Expression of various apoptotic and antiapoptotic proteins after a sublethal dose of Jo-2. **D) **Expression of BAD and p-BAD after a sublethal dose of Jo-2. **E) **Expression of various survival pathways after a sublethal dose of Jo-2.

To determine whether ILK may play a role in the regulation of hepatocyte survival from apoptosis inducing stimuli, we determined the sensitivity of mice lacking ILK to Fas-induced apoptosis. We injected ILK KO and control mice with a single intraperitoneal lethal dose (0.4 μg/g) of Jo-2. There was 50% mortality in the ILK KO (5/10) at 24 hours after Jo-2 injection, while all the controls died much faster than the ILK KO mice, showing 100% mortality (10/10) by 7 h after challenge whereas ILK KO mice were still alive at this time point (Figure 1A). Next we analyzed the effect of a sublethal dose of Jo-2 antibody (0.16 μg/g) on the survival of ILK KO and control mice. With this lower dose of Jo-2, there was 20% mortality (2/10) in the ILK KO mice while there was 70% mortality (7/10) in control mice by 24 h (Figure 1A). These data suggested that genetic ablation of ILK from hepatocytes protected the mice against Fas-induced apoptosis. We then evaluated the degree of hepatocellular damage in ILK KO and control mice in response to the sublethal dose of Jo-2. Histological examination of liver samples obtained at 6 h after sublethal dose of Jo-2 showed a higher degree of liver injury and the presence of parenchymal hemorrhages in control mice but not in ILK KO mice (Figure 1B). The different response to Jo-2 observed in ILK KO and control mice could be attributable in part to reduced hepatic expression of Fas receptor, because the basal levels of Fas as determined by Western blotting was lower in the livers of the ILK KO liver (Figure 1C). The expression was also lower in the hepatocytes isolated from ILK KO mice compared to WT mice (Figure 1C). Thus, it is likely that ILK regulates the expression of Fas receptor. Similarly, TUNEL assay of the liver sections demonstrated more abundant apoptotic nuclei in control mice than in ILK KO mice. Activation of capase3/7 was also higher in the control mice than ILK KO mice at 6 and 12 h after Jo-2 administration. In addition, expression of cleaved caspase 3 and PARP were also higher in the control than the ILK KO mice at both 6 and 12 h after a sublethal dose of Jo-2 (Figure 2A, B and 2C).

### Mechanism of protection of ILK KO mice against Jo-2 induced hepatic failure

We looked at the protein expression of various anti apoptotic proteins involved in Fas-induced apoptosis. Bcl-2 family proteins inhibit apoptosis induced by variety of stimuli, including Fas mediated apoptosis [1,14,15]. We assessed the expression of the antiapoptotic protein Bcl-xL and Bcl-2 by Western blotting at 0, 6 and 12 h after the injection of sublethal dose of anti-Fas antibody (Figure 2C). Bcl-xL and Bcl-2 proteins levels were decreased in the liver of control mice treated with Jo2; however, in ILK KO mice Bcl-xl and Bcl-2 protein levels were maintain in response to a sublethal dose of Jo-2 (Figure 2C). The ILK KO mice also had higher expression of Bcl-2 at basal levels (Figure 2C). We also looked at the protein expression of Bcl-2-associated death promoter (BAD) after Jo-2 administration. Dephosphorylated BAD forms a heterodimer with Bcl-2 and Bcl-xl, inactivating them, and thus allowing Fas-triggered apoptosis to take place. BAD phosphorylation is thus anti-apoptotic, and BAD dephosphorylation is pro-apoptotic [1]. In the control mice the BAD levels did not change before and after Jo-2 administration but there was an induction of BAD after Jo-2 administration in the ILK KO mice (Figure 2D). The expression of p-BAD which is antiapoptotic was higher in the ILK KO mice after JO-2 administration as compared to the control mice. The basal level of p-BAD was also higher in the ILK KO mice as compared to the controls (Figure 2D). Expression of p-BAD in control was barely detectable at basal levels (Figure 2D).

To understand the molecular events underlying the resistance of ILK KO mice to Jo-2 induced apoptosis, we examined the activation of several survival pathways known to be involved in cytoprotection against Fas-induced apoptosis. We investigated phosphorylation of Akt, Erk1/2, and NFκB activation which are known to be involved in cytoprotection against Fas-induced apoptosis [1,12,16,17]. There was an induction of both total and p-Akt after Jo-2 administration both in ILK KO and control mice at 6 and 12 h after Jo-2 administration (Figure 2E). The induction was more enhanced in the ILK KO mice than the controls at 6 h after Jo-2 administration (Figure 2E). Basal level of p-Akt was also higher in the ILK KO mice as compared to the controls. Levels of p-Erk1/2 levels at 6 h decreased after Jo-2 administration in the controls while they remain stable in the ILK KO mice (Figure 2E). Levels of total ERK were also slightly lower in the WT than ILK KO. Also, levels of the NFkB subunit p65 go down after Jo-2 in the control mice at 6 h while they were upregulated in the ILK KO mice. The basal level of p65 was also higher in the ILK KO mice as compared to the controls (Figure 2E). These studies suggest that the survival signaling machinery is upregulated in the ILK KO as compared to the controls before and after Jo-2 administration.

### Phosphorylation of ERK1/2 and NFκB activation are primarily responsible for protecting ILK KO hepatocytes from apoptosis

**Figure 3:**
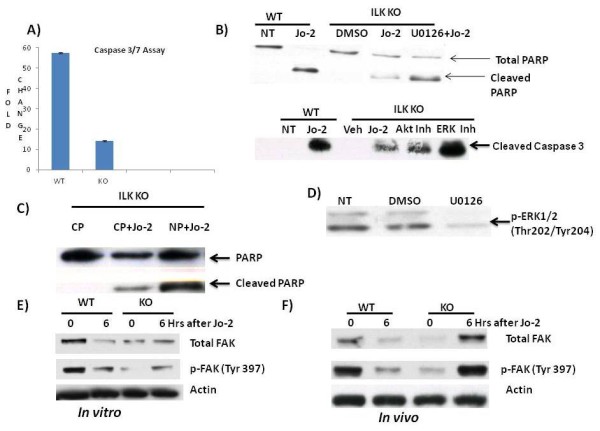
**ILK KO hepatocytes are protected against Jo-2 induced apoptosis in vitro**. **A) **Caspase 3/7 activity at 6 h after treatment of WT and ILK KO hepatocytes with Jo-2 (0.5 μg/ml) and Actinomycin D (0.05 μg/ml). Fold change is the ratio of luminescence value of treatment group with its corresponding no treatment group. **B) **Effect of ERK1/2 inhibition using a MEK inhibitor U0126 (20 μM). Representative Western blots of cleaved caspase and PARP 6 h after Jo-2, vehicle or Jo-2+inhibitor administration. (Akt Inh: Akt inhibitor LY-294002, ERK Inh: ERK inhibitor U0126) **C) **Representative Western blots showing inhibition of phosphorylation of ERK1/2 by U0126 in ILK KO hepatocytes after 6 h after treatment with U0126. **D) **Representative Western blots of PARP after inhibition of NFκB using a synthetic peptide 6 h after treatment with Control peptide (CP), CP+Jo-2 and NP+Jo-2 (NFκB peptide). (CP: control peptide, NP: NFκB peptide). **E) **Total FAK and p-FAK at 0 and 6 h after Jo-2 administration in vitro. **F) **Total FAK and p-FAK at 0 and 6 h after Jo-2 administration in vivo.

Consistent with our in vivo data, hepatocytes isolated from ILK KO mice were resistant to Jo-2 and Actinomycin D induced apoptosis (Figure 3A). Our in vivo data suggest that increase in survival pathways like Akt, Erk1/2 and NFκB plays a role in affording this protection. We used pharmacological inhibitors for Akt and Erk1/2 and peptide inhibitor for NFκB. Inhibition of Erk1/2 and NFκB led to increased susceptibility of ILK KO hepatocytes to Jo-2 and Actinomycin D induced apoptosis (Figure 3B and 3C). Pharmacological inhibitor against ERK1/2 was effective in downregulating the phosphorylation of ERK (Figure 3D). Inhibition of Akt did not have any effect (Figure 3B). Thus, NFκB and Erk1/2 but not Akt seem to be involved in affording protection to ILK KO hepatocytes to Jo-2 and actinomycin D induced apoptosis.

### Focal adhesion kinase signaling

Focal adhesion kinase is another enzyme associated with integrin signaling [18,19]. We looked into the possibility of FAK signaling compensating for the loss of ILK signaling. Genetic removal of ILK led to lower expression of FAK in the whole liver as well as hepatocytes isolated from the ILK KO mice (0 h of Figure 3E and 3F). Activation of FAK as a result of tyrosine phosphorylation at 397 residue was also lower in the whole liver as well as hepatocytes of the ILK KO mice (0 h of Figure 3E and 3F). Interestingly, administration of Jo-2 both in vivo and in vitro resulted in an increase in total as well as activated FAK in the ILK KO mice (Figure 3E and 3F). The WT mice on the other hand showed downregulation of total and activated FAK after Jo-2 administration both in vivo and in vitro. Several studies have shown the protective role of FAK in apoptosis [20-22]. Thus, upregulation of FAK signaling in the ILK KO mice after Jo-2 administration may also be playing an important role in protection against Jo-2 induced apoptosis. Interventional studies will provide a better understanding of the role of FAK signaling in Jo-2 induced apoptosis in absence of ILK signaling.

## Discussion

In this study we show that ILK is plays a regulatory role in Fas mediated apoptosis. We present evidence that hepatocyte specific ILK KO mice are resistant to Fas-induced apoptosis both in vivo and in vitro. Furthermore we show that apoptotic injury in the ILK KO mice is associated with an increase in antiapoptotic genes like Bcl-xl and Bcl-2. Investigation of the mechanism behind this protection revealed reduced expression of the Fas receptor in the ILK KO mice. However, the lower expression of Fas receptor in the ILK KO mice is not the only mechanism that could afford that much protection. Thus, we looked at the other possibilities that might also contribute to this protection. The survival program of ILK is well established and includes primarily activation of PI3K/Akt, ERK1/2 and NFκB pathway [6,7,23-25]. In agreement to these studies we found induction of PI3K/Akt, ERK1/2 and NFκB not only after Jo-2 administration but also at basal levels in the ILK KO mice. We then used a well described in the literature in vitro system of studying hepatocyte apoptosis using Jo-2 and Actinomycin D. Pharmacological inhibition of ERK using U0126 and peptide inhibition of NFκB pathway led to increased susceptibility of ILK KO hepatocytes to Jo-2 induced apoptosis in hepatocyte cultures, suggesting that ERK and NFκB pathways but were the signaling mediators for ILK in this process. Inhibition of Akt using PI3K inhibitor LY-294002 did not affect the degree of apoptosis in ILK KO hepatocytes. Together the data suggests that reduced expression of FAS receptor in the ILK KO mice along with persistent upregulation of survival signals like ERK1/2 and NFκB signaling is the mechanism behind protection of ILK KO mice against Jo-2 induced liver failure.

It should be noted that our results differ to previously published literature where upregulation of ILK in mammary epithelial cells protects against apoptosis [26]. It is conceivable that ILK may be promoting apoptosis in the liver while it has a completely opposite role in the mammary glands. Also, genetic elimination of a protein results in many adaptive changes in the organ. It is likely that genetic removal of ILK from the liver results in adaptive changes in the liver that make them resistant to apoptosis. Liver and mammary gland tissues also have different life cycles. Differentiation of liver tends to be stable through life whereas mammary glands undergo dramatic changes in their differentiation both due to hormonal cycles as well as during pregnancy.

A relevant question is why genetic ablation of ILK led to increased activation of these survival pathways? Our current studies as well as those we recently published [10,27-29] suggest that ILK mediated signaling plays a regulatory role the balance between proliferation and apoptosis in hepatocytes. Previous studies from our laboratory have also shown that in situations in which mitogenic signals to hepatocytes via EGFR or MET are suppressed, there is up-regulation of pro-apoptotic pathways and down-regulation of anti-apoptotic pathways [30,31]. The delicate balance between hepatocyte proliferation versus apoptosis underlies pathways leading to liver regeneration or liver failure. ILK has been shown to have many roles in tumor development, with studies describing different effects in different tumors based on tissue origin [24,25,32,33]. The signaling pathways by which ILK affects these phenomena were not clear. Our current studies with hepatocyte cultures show that at least in hepatocytes, the effects of ILK on hepatocyte survival are mediated via NFkB and ERK signaling. These signaling pathways also have well known effects on hepatocyte proliferation, and ILK seems to play a suppressive role in that regard (ILK KO hepatocytes have enhanced proliferation, [10,27].

## Conclusions

Here we report that genetic ablation of ILK from hepatocytes protects from Jo-2 induced apoptosis due to upregulation of survival signaling mainly ERK, FAK and NFκB signaling. The findings of this work provide a mechanistic interpretation of the ILK role in these processes and underscore the complex role of ILK and integrin signaling in control of proliferation, survival or death of hepatocytes.

## Competing interests

The authors declare that they have no competing interests.

## Authors' contributions

SD conducted the animal studies, collected tissues, performed Western blotting and wrote the manuscript. WM did the genotyping and gave technical assistance. AO performed the immunohistochemical staining. CW gave technical assistance. GM designed the study, examined histological and immunohistochemical staining, and reviewed the manuscript. All the authors have read and approved the final manuscript.
